# Resveratrol Inhibits HCoV-229E and SARS-CoV-2 Coronavirus Replication In Vitro

**DOI:** 10.3390/v13020354

**Published:** 2021-02-23

**Authors:** Sébastien Pasquereau, Zeina Nehme, Sandy Haidar Ahmad, Fadoua Daouad, Jeanne Van Assche, Clémentine Wallet, Christian Schwartz, Olivier Rohr, Stéphanie Morot-Bizot, Georges Herbein

**Affiliations:** 1 Pathogens & Inflammation/EPILAB Laboratory, EA 4266, Université de Franche-Comté, Université Bourgogne Franche-Comté (UBFC), 25030 Besançon, France; sebastien.pasquereau@univ-fcomte.fr (S.P.); zeina.nehme@univ-fcomte.fr (Z.N.); sandyhaydar@outlook.com (S.H.A.); 2 Lebanese University, P.O. Box 6573/14 Badaro Museum, Beirut, Lebanon; 3 FMTS, EA7292, Université de Strasbourg, IUT Louis Pasteur, 67300 Schiltigheim, France; f.daouad@unistra.fr (F.D.); jvanassche@unistra.fr (J.V.A.); clementine.wallet@unistra.fr (C.W.); schwartz.christian@unistra.fr (C.S.); olivier.rohr@unistra.fr (O.R.); 4 Apex Biosolutions, 25000 Besançon, France; smorot@apexlabo.com; 5 Department of Virology, Besancon University Hospital, 25030 Besançon, France

**Keywords:** SARS-CoV-2, HCoV-229E, resveratrol, coronavirus, viral inhibition

## Abstract

A novel coronavirus, severe acute respiratory syndrome coronavirus 2 (SARS-CoV-2), emerged in China at the end of 2019 causing a large global outbreak. As treatments are of the utmost importance, drug repurposing embodies a rich and rapid drug discovery landscape, where candidate drug compounds could be identified and optimized. To this end, we tested seven compounds for their ability to reduce replication of human coronavirus (HCoV)-229E, another member of the coronavirus family. Among these seven drugs tested, four of them, namely rapamycin, disulfiram, loperamide and valproic acid, were highly cytotoxic and did not warrant further testing. In contrast, we observed a reduction of the viral titer by 80% with resveratrol (50% effective concentration (EC50) = 4.6 µM) and lopinavir/ritonavir (EC50 = 8.8 µM) and by 60% with chloroquine (EC50 = 5 µM) with very limited cytotoxicity. Among these three drugs, resveratrol was less cytotoxic (cytotoxic concentration 50 (CC50) = 210 µM) than lopinavir/ritonavir (CC50 = 102 µM) and chloroquine (CC50 = 67 µM). Thus, among the seven drugs tested against HCoV-229E, resveratrol demonstrated the optimal antiviral response with low cytotoxicity with a selectivity index (SI) of 45.65. Similarly, among the three drugs with an anti-HCoV-229E activity, namely lopinavir/ritonavir, chloroquine and resveratrol, only the latter showed a reduction of the viral titer on SARS-CoV-2 with reduced cytotoxicity. This opens the door to further evaluation to fight Covid-19.

## 1. Introduction

Renowned as rapidly evolving viruses, human coronaviruses (HCoVs) are positive-stranded RNA pathogens that encompass seven different strains divided into two genera: alphacoronavirus (alphaCoV) and betacoronavirus (betaCoV) [1]. Whilst alphaCoV includes the lowly pathogenic HCoV-229E and HCoV-NL63, betaCoV ramifies into the more pathogenic HCoV-OC43 and HCoV-HKU1 of the A lineage and the highly pathogenic severe acute respiratory syndrome coronavirus (SARS-CoV), SARS-CoV-2 and the Middle East respiratory syndrome coronavirus (MERS-CoV) of the B and C lineage, respectively [2,3]. HCoV-229E, HCoV-NL63, HCoV-OC43 and HCoV-HKU1 are endemic in human populations, accounting for 15–30% of mild, self-limiting respiratory upper infections, with a greater incidence of lower respiratory tract infection in immunocompromised individuals [4]. Both SARS-CoV, emerging in 2002 with a mortality rate of 9.6%, and MERS-CoV, responsible for the 2012 outbreak with a death rate of 35.5%, are linked to acute respiratory distress syndrome (ARDS) potentially engendering a chronic reduction in lung function, arrhythmia or death [5]. Emerging in December 2019 from the city of Wuhan in China, SARS-CoV-2, the etiological agent behind coronavirus disease 2019 (Covid-19), is considered the cause of a major public health emergency after its large global outbreak [6]. Although causing milder symptoms compared with SARS-CoV and MERS-CoV, SARS-CoV-2 can result in severe symptoms with multiple organ complications and hyperinflammation, leading ultimately to death in some individuals [7].

Despite the fact that SARS-CoV-2 vaccines have been quickly developed [8], no definitive therapy has been shown to be effective to date against SARS-CoV-2 and therefore new treatments are actively needed [9]. Despite tremendous efforts to develop new drugs, it is well-known that this process might take years to be clinically approved and reach the market [10]. In this context, repurposing of existing drugs appears to be an effective drug discovery platform that is superior in terms of cost and time compared with de novo drug discovery, as rapid assessment of in vitro and in vivo efficacy is conceivable [11]. This is reinforced by the fact that agents previously shown to inhibit the in vitro replication of other members of the coronavirus family, for instance HCoV-229E, could be potential candidates to inhibit the replication of betacoronaviruses such as SARS-CoV, MERS-CoV and SARS-CoV-2 [12].

In the light of what has been mentioned previously, we screened seven drugs already approved for other pathologies against the alphacoronavirus HCoV-229E. On the assumption that an inhibitory effect against HCoV-229E by one of these drugs will be found, results will be extrapolated and assessed in the context of SARS-CoV-2-infected cell cultures.

Among the seven compounds tested, valproic acid (VPA), disulfiram, rapamycin and loperamide have been previously shown to target coronavirus infection [13]. Briefly, VPA is a branched short-chain fatty acid commonly used in the management of neurological disorders [14]. This histone deacetylase inhibitor was projected as a potential candidate for drug repositioning as the viral SARS-CoV-2 protein NSP5 was found to interact with HDAC2 [15]. Disulfiram, an approved alcohol-aversive drug, was shown to inhibit the papain-like proteases of both MERS-CoV and SARS-CoV [16]. The mTOR inhibitor rapamycin, a drug used as an immunosuppressant in transplant patients, was reported to reduce MERS-CoV infection by over 60% in vitro [17] by suppressing viral protein expression and virion release [18]. Alternatively, the antidiarrheal opioid receptor agonist loperamide showed potential antiviral activity against SARS-CoV-2 [11], as well as MERS-CoV, SARS-CoV and HCoV-229E, although the exact mechanism of action is unknown [19].

Being an aspartate protease inhibitor, lopinavir is an extensively studied antiviral drug used in the management of human immunodeficiency virus (HIV) infection in combination with ritonavir, the latter boosting the effects of lopinavir through potent cytochrome P450 3A4 inhibition [20]. Lopinavir was shown to be an inhibitor of broad-spectrum coronaviruses by blocking the replication of HCoV-229E, SARS-CoV and MERS-CoV with a mean 50% effective concentration (EC50) ranging from 6.6 to 17.1 μM, in contrast to ritonavir, which showed no selective anti-CoV activity [19]. SARS-CoV patients treated with a combination of lopinavir/ritonavir and ribavirin experienced less ARDS with a lower death rate compared with historical controls who received ribavirin and corticosteroids [21]. Randomized controlled trials were conducted to assess the efficacy of lopinavir/ritonavir with or without ribavirin in SARS-CoV-2 patients [22,23].

Used originally as treatment for or prevention of malaria, the 4-aminoquinoline drug chloroquine has been repurposed and successfully employed to target some immunological and rheumatological diseases, for instance systemic lupus erythematosus and rheumatoid arthritis [24]. In the setting of viral infections, multiple mechanisms underlie the effectiveness of chloroquine, prominently chloroquine-mediated alkalinization of phagolysosomes and/or viral entry and protein glycosylation inhibition [25]. Potential therapeutic benefits of chloroquine have been demonstrated in coronavirus infections including HCoV-229E, HCoV-OC43, SARS-CoV and SARS-CoV-2 [26]. Indeed, chloroquine inhibits HCoV-229E replication in Huh7 cells with an EC50 of 3.3 µM [19]. Moreover, pre and post treatment of Vero E6 cultures with chloroquine demonstrated antiviral activity against SARS-CoV-2 with an EC50 and EC90 of 1.13 μM and 6.90 μM, respectively [27]. In vivo, chloroquine administration was shown to inhibit SARS-CoV-2-related pneumonia, promote a virus negative conversion and reduce the disease course [28]. Based on these encouraging results, more than twenty in vivo clinical trials are in progress to evaluate chloroquine’s effectiveness against SARS-CoV-2 [29]. Nevertheless, the efficiency of chloroquine against Covid-19 remains highly controversial [30].

Belonging to the polyphenols stilbenoids group, resveratrol is a well-known nutraceutical with a multitude of biological properties and therapeutic applications [31]. Among the latter, resveratrol exhibits an anti-tumor activity that has been confirmed in in vitro and in vivo studies, where resveratrol modulates a divergent set of transcription factors, upstream kinases and intracellular signaling pathways, thus mediating its inhibitory effect on the initiation, promotion and progression of carcinogenesis [32]. In addition, resveratrol exhibits anti-aging and potent antioxidant properties [33], as well as an anti-inflammatory activity, where resveratrol was shown to reduce the secretion and expression of inflammatory factors [34]. Besides, resveratrol has been reported to exhibit a potent anti-viral activity not only against RNA viruses such as influenza virus [35], rhinovirus [36], rotavirus [37], Zika virus [38] and MERS-CoV [39], but also against a few DNA viruses including poxvirus [40] and polyomavirus [41]. In the context of anti-MERS-CoV activities in vitro, resveratrol was shown to inhibit the viral nucleocapsid protein translation and MERS-CoV-induced caspase 3 cleavage, which can promote cell survival and reduce virus-induced apoptosis [39]. Interestingly, resveratrol increases the expression of ACE2 on the cell surface, which has been previously reported to be beneficial for SARS-CoV-2 patients [42,43]. In fact, low levels of ACE2 are observed in Covid-19 patients with poor prognosis and hyperinflammatory storm [44]. In addition, resveratrol is a known activator of sirtuin 1 (SIRT1), which downregulates the expression of AT1R of the PRR/ACE/AngII/AT1R axis and enhances the ACE2/Ang 1-7/MasR axis [45]. Increase in the ACE2/Ang1-7/MasR axis has been reported to reduce hyperinflammation in Covid-19 patients [44]. In the present study, we assessed the anti-coronavirus activity of the seven abovementioned drugs with special emphasis on the impact of resveratrol on HCoV-229E and SARS-CoV-2 compared to lopinavir/ritonavir and chloroquine.

## 2. Materials and Methods

### 2.1. Viruses and Reagents Preparation

HCoV-229E was isolated from nasal and throat swabs collected from a man with mild upper respiratory illness (Human Coronavirus 229E ATCC VR-740) and propagated using MRC5 cells (RD Biotech, Besançon, France). HCoV-229E stock virus (5 log PFU/mL) was prepared in MRC5 cells in DMEM media supplemented with 10% FCS. SARS-CoV-2 strain was provided by the Institut Pasteur, Paris (strain BetaCov/France). Compounds were sourced from Sigma-Aldrich (St-Louis, MO, USA, resveratrol, chloroquine diphosphate), Pfizer, (New York, NY, USA, rapamycine), Sanofi (Paris, France, valproic acid or VPA, disulfiram), Abbott (Chicago, IL, USA, lopinavir/ritonavir) and Sandoz (Holzkirchen, Germany, loperamide). The stocks were prepared with DMSO (200 mM resveratrol, 50 mM lopinavir/ritonavir, 1 mM rapamycin, 2.5 mM VPA, 10 mM loperamide, 10 mM disulfiram) or with DMEM media (1 mM chloroquine diphosphate).

### 2.2. Viral Replication Inhibition Assay

To evaluate the effect of compounds in vitro, MRC5 cells were treated with compounds diluted in culture media at time of infection by HCoV-229E at multiplicity of infection (MOI) = 0.01 (low titer) or 1 (high titer). Antiviral compounds were maintained with the virus inoculum during the 2 h incubation period. The inoculum was removed after incubation, and the cells were overlaid with culture media containing diluted compounds. After 48 h of incubation at 37 °C, supernatants were collected to quantify viral loads by plaque forming unit (PFU) assay as previously described [46]. Briefly, MRC5 cells were infected with the supernatant for 2 h and then overlaid with culture media containing agarose (1%). Cells were incubated for 48 h. Plaque formation was observed under a light microscope. Cells were stained with MTT for 1 hour and plaque forming units were counted. Four-parameter logistic regression (IC50 Toolkit IC50.org) was used to fit the dose–response curves and determine the 50% effective concentrations (EC50) of the compounds that inhibit viral replication by 50%. Vero E6 cells were treated with compounds at time of infection by SARS-CoV-2 (BetaCoV/France, Institut Pasteur). The viral inoculum was removed after incubation and the cells were overlaid with fresh media containing the compound. After 48 h of incubation at 37 °C, supernatants were collected to quantify viral loads. Viral RNA was extracted using a guanidinium thiocyanate buffer and magnetic silica beads. Finally, RNA was detected for the SARS-CoV-2 envelop (E) gene using one step qRT-PCR (TaqMan Fast Virus 1-Step Master Mix, Applied Biosystem) and the primers designed by Corman et al. [47]. Briefly, the SARS-CoV-2 envelop gene was amplified using E_Sarbeco_F 5′-ACAGGTACGTTAATAGTTAATAGCGT-3′ and E_Sarbeco_R 5′-ATATTGCAGCAGTACGCACACA-3′ primers. Detection of amplification was done using a specific SARS-CoV-2 envelop probe (E_Sarbeco_P1) 5′-FAM-ACACTAGCCATCCTTACTGCGCTTCG-BHQ1-3′.

### 2.3. Cytotoxicity Assays

Cytotoxicity of selected compounds was evaluated in MRC5 cells using MTT assay. Briefly, cells were treated with compounds for 48 h. Culture media was replaced and cells were incubated with MTT (1.2 mM, Life Technologies, Eugene, OR, USA) for 2 h. Formazan crystals were dissolved by addition of acidic isopropanol and cell viability was measured by a spectrophotometer (Bio-Rad, Hercules, CA, USA). Cytotoxicity of compounds on Vero E6 cells was measured using several cytotoxic assays, including a WST-1 assay (Roche, Basel, Switzerland). The cytotoxic concentration 50 (CC50) is the concentration of compound that reduced cell viability by 50%. This was calculated using four-parameter logistic regression (IC50 Toolkit IC50.org).

Selectivity index (SI) was calculated by dividing CC50 by EC50 for each compound and virus tested. The selectivity index is an indicator that measures the window between cytotoxicity and antiviral activity. A high selectivity index reflects a low toxicity at concentrations that show an effective antiviral activity. It also indicates that there is an important range of concentrations showing an effective antiviral effect with minimal toxicity. Conversely, a low selectivity index would indicate an important toxicity for concentrations with effective antiviral activity.

## 3. Results

**Figure 1:**
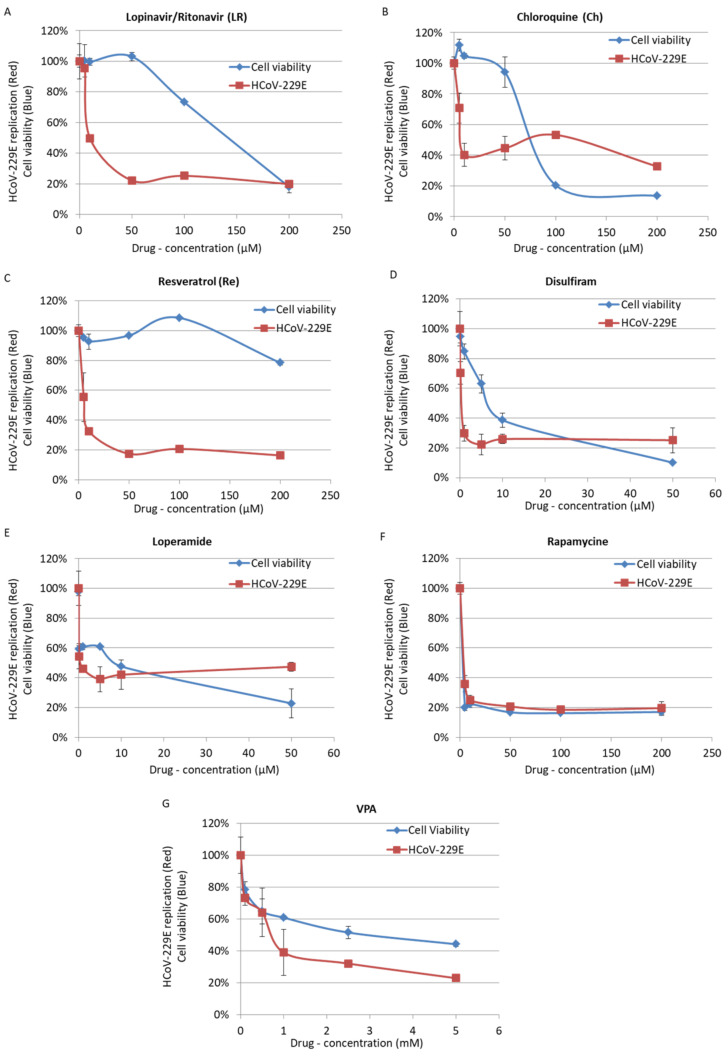
Inhibition of replication of high HCoV-229E viral load (Red), measured by plaque forming unit (PFU) assay, coupled with MTT toxicity assay (Blue) for Lopinavir/Ritonavir (**A**), Chloroquine (**B**), Resveratrol (**C**), Disulfiram (**D**), Loperamide (**E**), Rapamycin (**F**) and valproic acid (VPA) (**G**). Cells were treated by compounds at the time of infection with HCoV-229E (1 multiplicity of infection (MOI)). PFU and MTT assays were performed after 48 h. Uninfected cells were used for normalization of MTT assay. Untreated cells infected with HCoV-229E were used for normalization of PFU assay.

VPA, disulfiram, rapamycin and loperamide showed cytotoxicity on MRC5 cells with a CC50 of 4 mM, 8.4 µM, 2.5 µM, and 10.1 µM, respectively (Table 1, Figure 1). In addition, rapamycin and VPA display a narrowed selectivity index (SI) of 1.09 and 2.98, respectively (Table 1, Figure 1). By contrast, lopinavir/ritonavir, chloroquine and resveratrol were found to inhibit HCoV-229E replication in MRC5 cells with low cytotoxicity and an SI ranging from 11.6 to 45.6 (Table 1), and were tested further.

**Figure 2:**
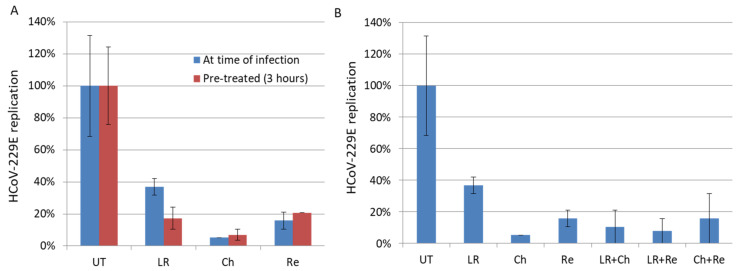
Inhibition of replication of low HCoV-229E viral load (0.01 MOI) by drugs. (**A**) No pretreatment (Blue) or 3-h pretreatment of cells (Red). (**B**) Combination of drugs. The cells were treated with Lopinavir/Ritonavir (LR), Chloroquine (Ch), Resveratrol (Re) or left untreated (UT) for 48 h. The viral replication was measured by PFU assay.

In line with previous reports [19], we observed an EC50 of 8.81 µM for lopinavir on HCoV-229E-treated MRC5 cells. We observed an antiviral effect of 50 µM lopinavir/ritonavir against high titers of HCoV-229E (MOI = 1) with approximately 80% inhibition of viral replication in vitro (Figure 1 and Table 1). Against low titers of HCoV-229E (MOI = 0.01), we observed a 63% inhibition of viral replication in cells treated with 10 µM of lopinavir/ritonavir (Figure 2). As combinational therapy of effective compounds against SARS-CoV-2 virus might convey additional benefits in terms of synergy and inhibitory concentration reduction [48], we therefore tested combinatory treatment of lopinavir/ritonavir with chloroquine and resveratrol. However, we did not observe a significant decrease in HCoV-229E in culture versus lopinavir/ritonavir when used alone (Figure 2B).

**Figure 3:**
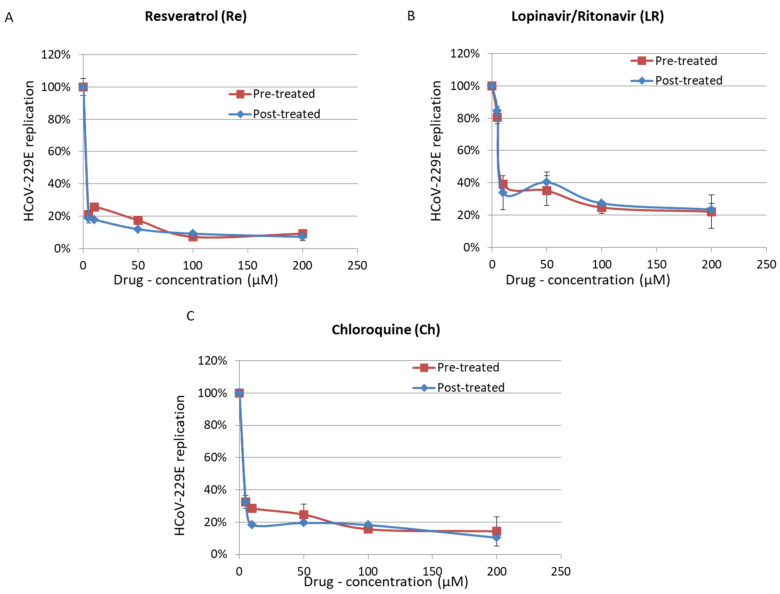
Inhibition of replication of high HCoV-229E viral load (1 MOI) by Resveratrol (**A**), Lopinavir/Ritonavir (**B**) and Chloroquine (**C**) in pre-treated (3 h, Red) or post-treated (4 h, Blue) MRC5 cells. Viral replication was measured by PFU assay after 48 h.

As reported previously, we observed that chloroquine inhibits HCoV-229E replication in MRC5 cells when added 3 h before infection but also when added at the time of infection (Figure 2). We also observed that chloroquine inhibits the replication of HCoV-229E in cells infected with a high titer of virus (EC50 of 5 µM; Figure 1). This is in agreement with the antiviral activity of hydroxychloroquine, a chloroquine derivative, with an EC90 of 5.47 µM in the context of SARS-CoV-2 infection [49]. We did not observe a synergistic effect of chloroquine used in combinatory treatments with lopinavir/ritonavir or resveratrol (Figure 2b). We also did not observe a significant difference in the effect of chloroquine when cells were treated before or after the infection (Figure 3). We observed some cytotoxicity of chloroquine on treated cells, with a CC50 of 67.9 µM (Figure 1 and Table 1).

**Figure 4:**
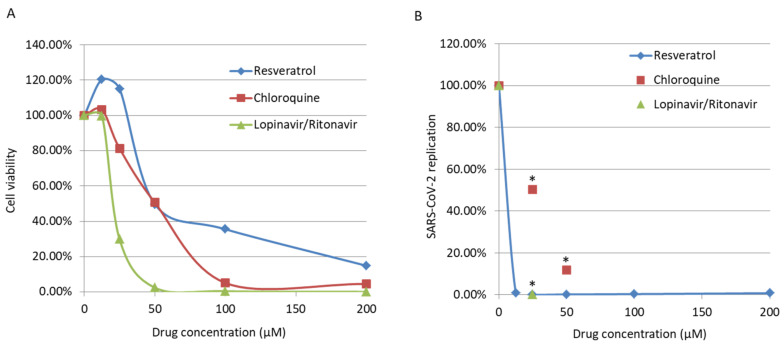
(**A**) Toxicity assay for Lopinavir/Ritonavir, Chloroquine and Resveratrol in Vero E6 cells, after 48 h. (**B**) Inhibition of severe acute respiratory syndrome coronavirus 2 (SARS-CoV-2) replication in Vero E6 cells treated with Resveratrol as measured by qRT-PCR after 48 h. * Cytotoxicity impaired the evaluation of the anti-SARS-CoV-2 effect of Lopinavir/Ritonavir and Chloroquine.

Here, we observed that resveratrol inhibits HCoV-229E replication in MRC5 cells infected with a high viral titer (MOI = 1), with an EC50 of 4.6 μM (Figure 1 and Table 1). We also observed an inhibition of replication in cells infected with a low viral titer (MOI = 0.01) and treated with resveratrol (Figure 2). We did not observe a significant difference in the effect of resveratrol when cells were treated before or after the infection (Figure 3). We did not observe any synergistic effect of resveratrol used in combination with lopinavir/ritonavir or chloroquine (Figure 2b). Resveratrol displayed a low toxicity in treated cells, with a CC50 of 210 µM and a selectivity index (SI) equal to 45.65 (Figure 1 and Table 1). Based on the results obtained for HCoV-229E, we performed testing for SARS-CoV-2 inhibition. Treatment of Vero E6 cells with lopinavir/ritonavir and chloroquine was toxic and did not allow for the study of their anti-SARS-CoV-2 effect (Figure 4a). The important cell lysis in culture prevented an accurate measure of SARS-CoV-2 replication, giving partial and unreliable results (Figure 4b). In contrast, the cytotoxicity of resveratrol on Vero E6 cells occurred at concentrations higher than 50 µM (Figure 4a). We observed that resveratrol inhibited SARS-CoV-2 replication by 3 logs at 25 µM (Figure 4b). The percentage of inhibition of SARS-CoV-2 ranges from 0 to 99.93% when increasing the concentration of resveratrol from 0 to 25 µM with an EC90 and EC50 of 11.42 µM and 10.66 µM, respectively (Figure 4). A very limited reduction in cell viability was also observed in Vero E6 cells treated with resveratrol at 50 µM, overall leading to a CC50 = 48.21 µM and an SI of 4.52. The inhibition of SARS-CoV-2 replication we observed in cells treated with resveratrol did not follow the reduction in cell viability and was a genuine anti-SARS-CoV-2 effect of resveratrol in vitro.

## 4. Discussion

We confirm the antiviral activity of two compounds (lopinavir/ritonavir and chloroquine) that have been reported previously to inhibit HCoV-229E replication in vitro. In addition, we show the antiviral effect of resveratrol on HCoV-229E and SARS-CoV-2 replication in vitro. In addition, resveratrol is the most powerful compound with an EC50 of 4.6 µM, a CC50 of 210 µM and an SI of 45.65, indicating that it could be efficiently used to fight HCoV-229E infection. While their SI is lower, lopinavir/ritonavir and chloroquine might show clinical potential. For instance, it has been observed that HIV patients might reach minimal lopinavir serum concentration at 9.4 μM (IQR 7.2–12.1 μM) upon twice daily treatment with 400 mg lopinavir and 100 mg ritonavir [50], which is in the same range of the EC50 against HCoV-229E virus in vitro (8.8 µM). The previously mentioned dosing regimen has been recommended by the by Chinese National Health Commission in the management of SARS-CoV-2 patients [51]. Nonetheless, no differences in time of clinical improvement, mortality at 28 days and detectable viral loads at various time points were reported between the lopinavir/ritonavir group and the standard-care group in a recent randomized, controlled, open-label trial [22]. Overall, a beneficial effect of lopinavir/ritonavir and chloroquine in Covid-19 patients is still highly debated.

We observed a high cytotoxicity of lopinavir/ritonavir and chloroquine on Vero E6 cells, which precluded the study of their anti-SARS-CoV-2 effect. In fact, several studies have already evaluated the anti-SARS-CoV-2 effect of drugs including lopinavir and chloroquine [48,49]. While most studies showed an antiviral effect in vitro for these two drugs that would warrant further investigation, they also indicated cytotoxicity of these drugs. The cytotoxicity we observed for lopinavir/ritonavir and chloroquine in the present study is in line with these previous reports [48,49].

In contrast to lopinavir/ritonavir and chloroquine, our testing for SARS-CoV-2 inhibition shows a genuine anti-viral effect of resveratrol. We observed a significant decrease in SARS-CoV-2 replication under non-cytotoxic doses of resveratrol up to 25 µM in vitro. To further confirm the anti-SARS-CoV-2 effect of resveratrol using an in vitro model closer to the in vivo pathophysiology, we will assess in future studies the anti-SARS-CoV-2 effect of resveratrol using organotypic nasal epithelial cultures, which might better reflect the pathophysiology of SARS-CoV-2 infection compared with the renal epithelial Vero E6 cells. During the revision process of our manuscript, new reports that showed the effect of resveratrol on SARS-CoV-2 replication became available. Yang et al. reported the inhibition of SARS-CoV-2 replication by resveratrol, with an EC50 of 4.48 µM, which is in line with our findings [52]. In a study yet to be published, Ter Ellen and colleagues showed that resveratrol and the structurally related molecule pterostilbene both inhibited the replication of SARS-CoV-2, especially in primary human bronchial epithelium cells [53].

Although resveratrol, which displays both antiviral and anti-inflammatory properties, could be an interesting treatment for Covid-19 patients [54], its use is limited by poor bioavailability. Multiple strategies have been employed to overcome the poor bioavailability of resveratrol in vivo [55], including a soluble formulation of trans-resveratrol [56] and its administration as a nasal spray [57]. Although administered doses vary widely between 10 mg and 5 g [58], previous pharmacokinetic studies showed that patients receiving a single 500 mg oral dose of resveratrol might reach an adequate plasma concentration to promote the pharmacological activities of 71.2 ± 42.4 ng/mL e.g., 0.31 µM, with a Tmax of 1.3 h [59]. In addition, nasal administration to expose the lungs to resveratrol reduced tumor volume and tumor multiplicity by 45% and 27%, respectively, in A/J mice with lung cancer [60]. In Covid-19 patients hospitalized in emergency units, the use of naso-tracheal administration of resveratrol might allow for direct delivery of the treatment into the lung and could provide maximum antiviral and anti-inflammatory efficiency.

In conclusion, further testing is needed to confirm whether resveratrol, either alone or in combination therapy, could be of use in a clinical setting. Finally, it is important to note that our results on SARS-CoV-2 inhibition by resveratrol are in vitro data and do not indicate that resveratrol can be used to treat Covid-19 until double-blinded clinical assays have been successfully performed.

## 5. Conclusions

We confirm the anti-HCoV-229E activity of two compounds, lopinavir/ritonavir and chloroquine, that have been previously reported to inhibit HCoV-229E replication in vitro. In addition, we show here the antiviral effect of resveratrol on HCoV-229E and SARS-CoV-2 replication in vitro. Resveratrol is the most powerful compound to fight HCoV-229E infection with an EC50 of 4.6 µM, a CC50 of 210 µM and an SI of 45.65. In addition, our testing for SARS-CoV-2 inhibition shows an anti-viral effect of resveratrol in vitro, which will be assessed in future studies in organotypic nasal epithelial cultures. This will better reflect the pathophysiology of Covid-19 infection and might lead to its use in a clinical setting, either alone or as part of a combined treatment against Covid-19.

## Figures and Tables

**Table 1:** The 50% effective concentration (EC50), cytotoxic concentration 50 (CC50) and selectivity index (SI) for the seven drugs tested for human coronavirus (HCoV)-229E replication in MRC5 cells.

Drug Tested	CC50 (µM)	EC50 (µM)	SI
Lopinavir/Ritonavir	102.5	8.8163	11.62619
Chloroquine	67.9	5	13.58
Resveratrol	210	4.6	45.65217
Rapamycin	2.5	2.28	1.096491
Disulfiram	8.4	0.1387	60.56236
Loperamide	10.1	0.094732	106.6166
VPA	4 mM	1.339 mM	2.987304

## Data Availability

The data presented in this study is available on request from the corresponding author.
